# Molecular and morphometric analysis of nominal *Brachidontes exustus* (Mollusca, Mytilidae) in Brazilian waters

**DOI:** 10.1590/1678-4685-GMB-2021-0247

**Published:** 2022-04-29

**Authors:** David B. Quintanilha, Flavio C. Fernandes, Caroline R. Guerra, Savio H. C. Campos, Laura I. Weber

**Affiliations:** 1 Universidade Federal Fluminense (UFF), Instituto de Estudos do Mar Almirante Paulo Moreira (IEAPM), Programa de Pós-Graduação em Biotecnologia Marinha, Arraial do Cabo, RJ, Brazil.; 2 Marinha do Brasil, Instituto de Estudos do Mar Almirante Paulo Moreira (IEAPM), Arraial do Cabo, RJ, Brazil.; 3 Universidade Federal do Rio de Janeiro (UFRJ), Instituto de Biodiversidade e Sustentabilidade, Macaé, RJ, Brazil.

**Keywords:** Brazil, Noronha, Amazon Barrier, Brachidontes, mDNA

## Abstract

*Brachidontes exustus* (Mollusca, Mytilidae) is mainly distributed in Central America, where it has been recognized as a _lataforma species. This study aimed to determine whether *B. exustus* extends beyond the Amazon Barrier and southward along the Brazilian West Atlantic coast. Mitochondrial genes coding for cytochrome-*c* oxidase, subunit I (COI) and 16S subunit of ribosomal _lataforma__ cid (16S rRNA) were analyzed with _lata parameters on Brazilian populations (Salvador, Arraial do Cabo and Fernando de Noronha) of scorched mussels previously recorded as *B. exustus*. Multivariate morphometric _latafor showed partial discrimination of species. Molecular _latafor confirmed *B. exustus* at Salvador, a population highly similar to Cartagena (Colombia), both belonging to the Atlantic Clade of the *B. exustus* complex. This fact adds evidence to the idea of the Amazon outflow as a semipermeable barrier. In the southeast of Brazil, *B. exustus* was not found; instead, *B. darwinianus* is the species represented at Arraial do Cabo (state of Rio de Janeiro), associated with brackish _lataf. Scorched mussels from Fernando de Noronha are most closely related to *B. puniceus* from Cape Verde with 4.4% differentiation. Demonstrating an independent evolutionary history since at least the beginning of the Pleistocene, its proposed new name is *B. noronhensis.*

## Introduction

The marine biota of the Brazilian coast belongs to the Guyanese Province (with Amapá; Barroso et al., 2016), to the Brazilian Province and the Argentinian Province (Briggs and Bowen, 2013). Species from oceanic islands are attributed to the Brazilian Province, represented mainly by tropical species (Briggs and Bowen, 2012; Barroso *et al*., 2016; Floeter et al., 2009). Although, Caribbean species reach the Brazilian Province, the degree of endemism in Brazil allowed it to be considered a distinct province. Two main barriers to dispersal have been identified along the Brazilian coast: the Amazon outflow in the Tropical Brazilian Province and the significant extension of sandy beaches found in the Argentinian Province (Floeter *et al*., 2009) both considered acting as selective barriers.

Along the Atlantic coast of South America, four species of *Brachidontes* have been cited so far: *B. exustus* (Linnaeus, 1758), *B. darwinianus* (d’Orbigny, 1846), *B. rodriguezii* (d’Orbigny, 1842) and *B. purpuratus* (Lamarck, 1819). Another species initially described as *Brachidontes solisianus* d’Orbigny, 1846, was transferred to the genus *Mytliaster* Monterosato,1883 (*Mytilaster solisianus*) by Huber (2010). The absence of shell ribbing and lack of marginal denticles were considered evidence for not belonging to the genus *Brachidontes* (Morton, 2012)*. M. solisianus* was considered by Trovant et al. (2016) the result of a vicariant event of *B. exustus,* mediated by the outflow of the Amazon River, after its expansion to the south of South America Atlantic waters during the Miocene. The genus *Brachidontes* (Swainson, 1840) originated in the Jurassic (Morton, 2012) and is a near-worldwide genus of scorched mussels living on natural and rigid artificial substrates of shallow waters (Aguirre et al., 2006). It is represented by _lataforma controlled by temperature, substrate, energy, water depth, and salinity (Aguirre *et al*., 2006; Trovant *et al*., 2013); and it has a wide variety of habitats, inhabiting brackish waters of river outlets, estuaries, mangrove swamps (Bennett et al., 2011; Morton, 2012) and marine coastal to open ocean (Rios and Barcellos, 1979; Lee and Foighil, 2004; Trovant *et al*., 2013, 2015, 2016).

The species *B. Exustus* was long considered the most widespread species, with a wide variety of habitats ranging from North Carolina, through Central and South America, to Argentina (Jensen and Harasewych, 1986; Rios, 1994). Based on shell morphology, Rios (1994) listed the species for this broad range, considering other described species of *Brachidontes* as synonyms (e.g. *B. domingensis and B. darwinianus*). *B. exustus* was recorded for the northeast coast of Brazil (Klappenbach, 1965), for the state of Rio de Janeiro (Quintanilha, 2017), and also for the oceanic islands of Fernando de Noronha (Lopes and Alvarenga, 1955; Rios and Barcellos, 1979) and Santa Helena (Rios, 1994). The lack of significant differences among shells from fossil and living specimens also led Aguirre et al. (2006) to believe that variations in form were only adaptations to different environmental conditions.

The type locality of *B. exustus* is Jamaica in the Caribbean Sea. However, the lack of morphological diagnostic characters to distinguish the species correctly made it necessary to use molecular markers to determine the taxonomic status of different ecotypes. Lee and Foighil (2004, 2005) and Bennett et al. (2011), using molecular markers for populations from Florida and the Caribbean Sea, concluded that *B. exustus* is a complex of species rather than a single species; identifying four distinct genetic clades: the Atlantic, the Gulf of Mexico, The Bahamas and Antilles clades (Lee and Foighil, 2004, 2005).

Trovant et al. (2013) enhanced the need to resolve the taxonomic status of the South American *Brachidontes* species and demonstrated by molecular markers the specific status of *B. darwinianus*, *B. rodriguezi* and *B. purpuratus* for the south western Atlantic. That distribution was found to be tightly correlated to sea-surface temperature and salinity. *B. darwinianus*, long considered as a synonym of *B. exustus,* has its type locality in Rio de Janeiro and is associated with subtropical and warm-temperate waters of southern Brazil and Uruguay, and it is known to be tolerant to low salinity waters of estuarine zones in lower mid-littoral areas (Tanaka, 2005; Aguirre et al., 2006; Trovant *et al*., 2013, 2016). This species is replaced further south by *B. rodriguezi* in the Gulf of San Mathias and is also characteristic of the warm-temperate region of Argentina (Trovant *et al*., 2013), with Rio Negro-San Blas, northern Patagonia, as type locality (Aguirre *et al*., 2006). *B. rodriguezi* is replaced by *B. purpuratus* (Lamarck, 1819) from Golfo Nuevo down to Tierra del Fuego in the cold temperate waters of the Magellan Province, extending northward along the Pacific coast into the Chile-Peru Biogeographic Province (Aguirre *et al*., 2006; Trovant *et al*., 2013, 2015). 

A recent study suggested that the distribution of *B. exustus* is restricted to the tropical regions not overpassing the Amazon Barrier (Trovant et al., 2016), which was supported by the absence of the species in collected areas of South America (Trovant *et al*., 2013, 2015, 2016). However, populations of scorched mussels _lataforma recorded as *B. exustus* in the Brazilian coast (Rios and Barcellos, 1979; Rios 1994; Quintanilha, 2017) must be re-evaluated. Therefore, we aimed to analyze those populations using molecular markers and shell morphology and to recover part of the phylogeographic pattern within the *B. exustus* complex.

## Material and Methods

### Sampling sites

**Figure 1 -:**
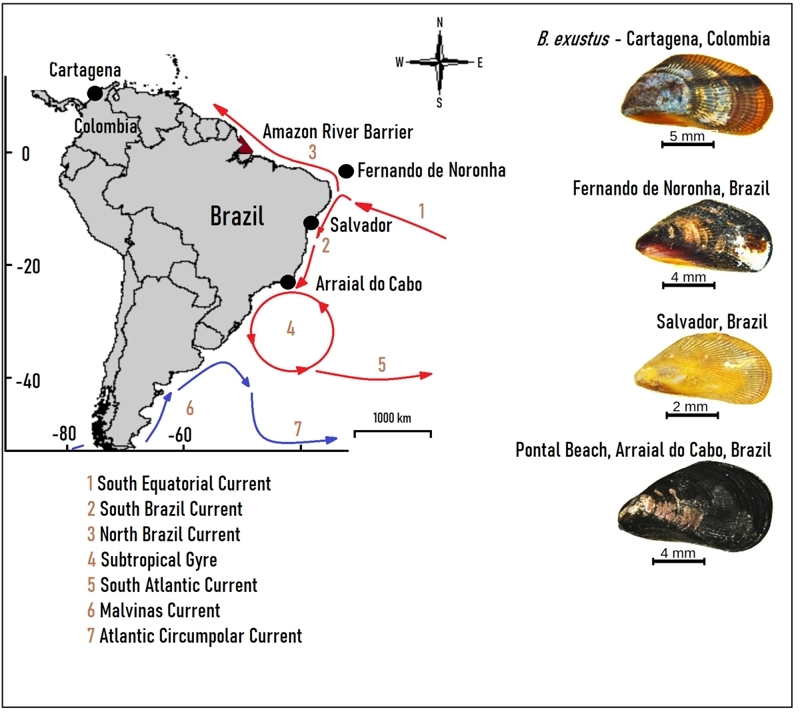
Sampling sites (black circles) showing scorched mussel morphotypes (right column). Major South Atlantic current systems are based on Muller-Karger et al. (2017): red arrows = warm waters; blue arrows = cold waters (1-7).

Scorched mussels identified previously by morphology as *B. exustus* were collected between October 2013 and November 2016 from Brazilian localities (Figure 1) as follows: 1. Scorched mussels (N = 75) were obtained from the open sea rocky shore of Cacimba do Padre Beach (3˚51’S, 32˚26’W), situated on the main island of Fernando de Noronha Archipelago (FN), at 545 km from the coast of Recife, State of Pernambuco. Salinities in this area were characteristic of marine waters (35.7-36.3), and with water temperatures varying from 26.5 °C to 29.2 °C (Santana et al., 2018). 2. Scorched mussels (N = 48) were also obtained from a stone wall within a mangrove swamp at the Todos os Santos Bay (12°48’S; 38°28’W) of Salvador, State of Bahia (SV). At this site, salinities varied from 31 to 36.2, and water temperatures ranged from 23 °C to 28 °C (Miranda et al., 2011). 3. Mussels were also collected from Arraial do Cabo, State of Rio de Janeiro. Scorched mussels (N = 75), previously nominated as *B. exustus*, were obtained from rocks in the outlet of a small brackish canal at Pontal Beach (PB) (22°56’46’’S; 42°01’49’’W), where salinities showed a wide range (28.7-36.8) with water temperatures of 22.7 °C - 22.9 °C (Quintanilha, 2017). At this site, from the rocky shore of Anjos Beach (AB) (22º58’42’’S; 42º01’12”W), Arraial do Cabo, were also collected individuals from *M. solisianus* to know its molecular pattern and rule out any *M. solisianus* that may pass within the nominal *B. exustus* samples. Marine water salinities in the area were at 36, and water temperatures vary significantly during the year (12 °C - 20 °C) (Valentin et al., 1987; Coelho-Souza et al., 2012). *Brachidontes exustus* (N = 48) from Cartagena Bay (10°24’N, 75˚31’W), Colombia, were also obtained from a mangrove area for comparison with the Brazilian populations. In this area, salinities may vary from 21 to 35.9, and water temperatures have been observed from 27.8 °C to 30.5 °C (Tosic et al., 2019).

### Molecular analysis

Total DNA was extracted from muscle tissue using the Wizard^®^ Promega commercial kit and the phenol/chloroform/proteinase-K protocol (Hoelzel, 1998). DNA amplifications of partial regions of the 16S rRNA gene (*16S*) and cytochrome-*c*-oxidase subunit I gene (*COI*) were conducted using polymerase chain reaction (PCR) and universal primers: 16sar-L, 5’-cgcctgtttatcaaaaacat-3’; 16sbr-H, 5’-ccggtctgaactcagatcacgt-3’ (Palumbi et al., 2002) and HCO2198, 5’-taaacttcagggtgaccaaaaaatca-3’; LCO1490, and 5’-ggtcaacaaatcataaagatattgg-3’ (Folmer et al., 1994). When amplification using universal primers failed, new primers were designed for the *COI* gene using sequences of scorched mussels obtained from GenBank and aligned with the Clustal W online tool. The designed primers were evaluated using the Integrated DNA Technologies (IDT) OligoAnalyzer tool. The primers designed for *B. puniceus* (Gmelin, 1791) from Cape Verde Archipelago (Cunha et al., 2011; see Table S1), BpunF: *5*’*-*cacccaggtaactttttgtt-3’, and BpunR: *5*’*-*cagcatagtaatacctccag-3’, allowed the amplification of the COI gene of scorched mussels from Fernando de Noronha. Amplifications used a 25 µL final volume with 1x reaction buffer, 3 mM MgCl2, 0.24 mM of each dNTP, 0.12% Triton-X-100, 0.4 µM of each primer, and 2 units of DNA polymerase for 1 µL of extracted or diluted DNA. This reaction was submitted to the following cycles using a thermocycler (Eppendorf Gradient mastercycler^®;^ SigmaAldrich, Germany): 1 cycle at 94 ºC for 4 min; 35 cycles at 92 ºC, 52 ºC, and 72 ºC for 1 min each; and a final cycle of 72 ºC for 7 min. DNA fragments were visualiszed after electrophoresis under UV light using ethidium bromide or the fluorescent Unisafe stain (Uniscience Corporation). PCR products were sequenced by Sanger Technology, edited with Chromas Pro, version 2.1.8 software, aligned with online tool Clustal W, and identity with other sequences from GenBank were obtained using BLAST: Basic Local Alignment of nucleotide sequences at the National Center for Biotechnology Information (NCBI).

Genetic data analysis

Haplotypes for each *locus* (*16S* and *COI*) were identified, and their frequencies (*f*_*x*_) were determined. The lack of *16S* sequences from *Brachidontes* species in GenBank did not allow comparisons with species of other localities at this *locus*. A maximum likelihood (ML) tree was constructed using Tamura-Nei distance (d) with MEGA, v. 6 software (Tamura et al., 2013), using all our samples and consensus sequences of main Lee and Foighil (2004, 2005)’ clades: Gulf of Mexico, Atlantic, The Bahamas A, The Bahamas B-Boca Chica, The Bahamas B-Biscayne, The Bahamas C, Antilles A and Antilles B clades. A consensus sequence of *Geukensia demissa* (Dillwyn, 1817) and sequences of *Mytilus edulis* Linnaeus, 1758, were used as outgroups. Bootstrap iterations (N = 1,000) were used for branch confidence in the ML tree. Time since divergence between species was calculated considering the mean rate of nucleotide substitution obtained by Marko (2002) for bivalve COI gene (1.21 Myr) and the conventional estimated rate of evolution in mitochondrial DNA (2% of sequence per Myr; Brown et al., 1979) between pairs of lineages, using t = ½*d*/*µ.* Haplotype networks were constructed using POPART software and the method of Templeton, Crandall and Sing (TCS) network (Clement et al., 2002). Pair-wise, Tamura-Nei distances were obtained among populations and species. Accession numbers of the sequences obtained from GenBank and obtained in this study are shown in Table S1.

### Morphometric analysis of shells

**Figure 2 -:**
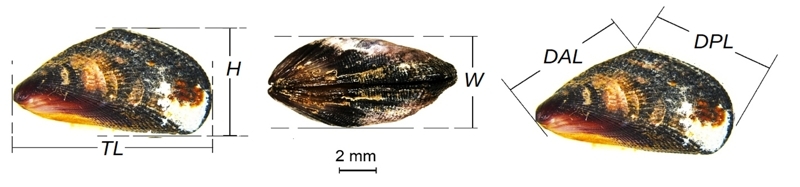
Linear shell dimensions obtained from scorched mussels: (TL) length, maximum distance between the umbone and the posterior margin of the shell; (H) height, the maximum distance between dorsal and ventral margins; (W) width, the maximum distance between valves; (DAL) anterior dorsal angle length, the maximum distance between the umbone and the dorsal angle; and (DPL) posterior dorsal angle length, the maximum distance between the dorsal angle and the posterior margin of the shell.

Morphometric analyses were based on five linear dimensions: total length (TL), height (H), width (W), anterior dorsal angle length (DAL), and posterior dorsal angle length (DPL) (Figure 2). All measurements with 0.01 mm precision were taken from mussels’ left valves with MT-00855 Uyustools Professional digital caliper. Shells were digitalized with a Leica M205-FA multifocal stereoscope microscope. All specimens and/or tissues were deposited in the Scientific Collection of the Instituto de Estudos do Mar Almirante Paulo Moreira (IEAPM), Marinha do Brasil, Arraial do Cabo, RJ, Brazil, under the following batch numbers: Batch# 3370 and Batch# CT 065 (Salvador, Brazil); Batch# CT 063 (Fernando de Noronha, Brazil); Batch# 3367 and Batch# CT 064 (Pontal Beach, Arraial do Cabo, Brazil); Batch# 3369 (*Brachidontes exustus*, Cartagena, Colombia). Relative measurements (rH, rW, rDAL, rDPL) were obtained by dividing each parameter by the TL. The new relative variables were evaluated for normal distribution using the Kolmogorov-Smirnov test (5% significance). Canonical Discriminant Analysis (CDA) was performed by grouping mussels into their respective populations (localities) and species (according to the molecular analysis). Cases were plotted in the two-dimensional space formed by the two canonical roots. Classification functions were used to evaluate the discriminant power for correct classification, first by population and second by species. The CDA was performed using Statistica^TM^ for Windows, version 7 Statsoft Inc.).

## Results

### Molecular analysis

*Scorched mussels from Salvador, State of Bahia*

**Figure 3 -:**
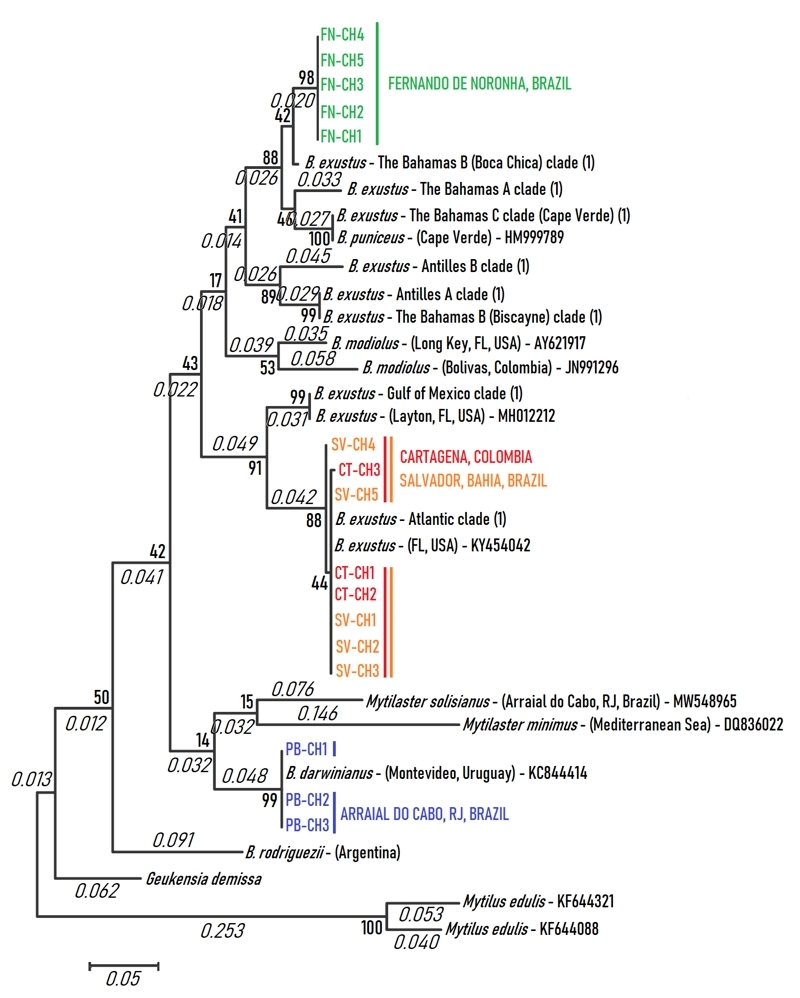
The maximum likelihood phylogenetic tree based on Tamura-Nei distances of *COI* nucleotide sequences, showing the phylogenetic positions of scorched mussels obtained in this study (in blue), branch length (in italics), and bootstrap branch confidence. Lee and Foighil (2004, 2005) clades of *B. exustus* complex, most represented by consensus sequences (1). For GenBank accession numbers, see Table S1

**Figure 4-:**
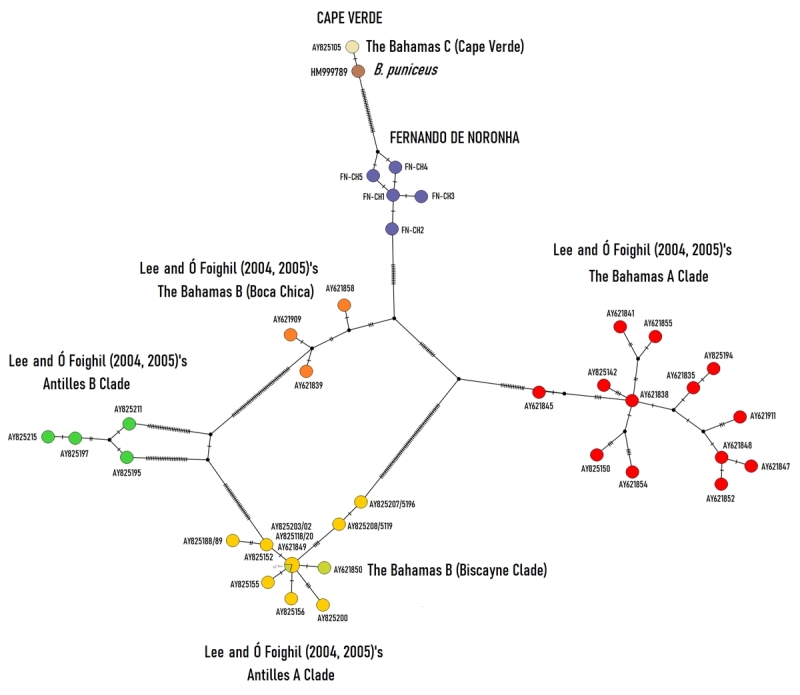
The TCS network showing *COI* haplotypes of scorched mussels from Salvador, Cartagena, and from taxa of *B. exustus* complex belonging to the Gulf of Mexico and Atlantic clades (Lee and Foighil, 2004, 2005). Haplotypes are represented by circles, the size of which is not proportional to haplotype frequency. GenBank accession numbers can be found in Table S1.

At both *loci* (*16S* and *COI*) Salvador scorched mussels were highly similar (N= 10, *16S*, *d* = 0.002 ± 0.001; *COI*, *d* = 0.001 ± 0.001) to *B. exustus* from Cartagena. Salvador shared with Cartagena the two most common haplotypes out of three at the *16S locus* (*f*_*SV-H1*_ = 0.600, *f*_*SV-H2*_ = 0.300). At *COI locus*, Salvador did not share with Cartagena (N = 3) the two most common haplotypes (*f*_*SV-CH1*_ = 0.500 and *f*_*SV-CH2*_ = 0.200) out of five, probably due to the low number of sequences obtained for Cartagena, caused by bacterial contamination. Both were very close genetically to the consensus sequence of the Atlantic Clade (*d* = 0.001 ± 0.001) and differing from the Gulf Clade in 7.5% (*d* = 0.075 ± 0.015; Figure 3). The haplotype network (Figure 4), which includes all members of Lee and Foighil (2004, 2005) clades (Atlantic and Gulf of Mexico), shows in detail where Salvador scorched mussels are situated within the Atlantic Clade.

*Scorched mussels from the Fernando de Noronha, State of Pernambuco*

**Figure 5 -:**
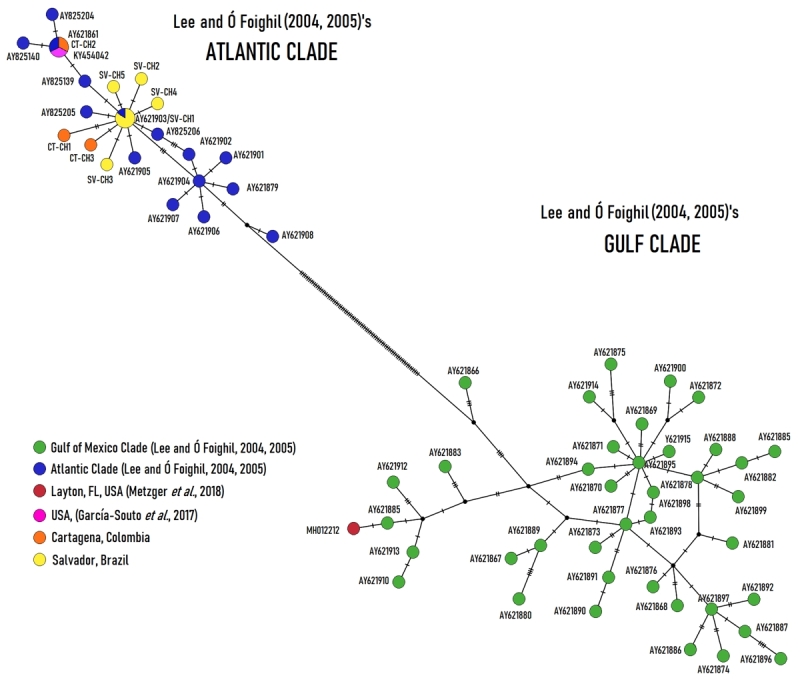
The TCS network showing *COI* haplotypes of scorched mussels from Fernando de Noronha and from taxa of *B. exustus* complex belonging to The Bahamas and Antillean clades (Lee and Foighil, 2004, 2005). Haplotypes are represented by circles, the size of which is not proportional to haplotype frequency. GenBank accession numbers can be found in Table S1.

Fernando de Noronha scorched mussels were highly distant from all scorched mussels analyzed in this study as revealed by both *loci* (to *B. exustus* from Salvador and Cartagena, *16S*: *d* = 0.166 ± 0.020, *COI*: *d* = 0.139 ± 0.021; and to the species from Pontal Beach, *16S: d* = 0.139 ± 0.019, *COI*: *d* = 0.139 ± 0.021). The group of mussels from Fernando de Noronha showed two haplotypes at *16S locus* with the most common haplotype in high frequency (N = 22, *f*_*FN-H1*_ = 0.954); and showed five haplotypes at *COI locus* with the most common one also in high frequency (N = 15, *f*_*FN-CH1*_ = 0.733). It was most closely related to *B. puniceus,* showing a distance of 4.4% (*d* = 0.044 ± 0.011), followed by The Bahamas A consensus sequence (*d* = 0.067 ± 0.014). Fernando de Noronha scorched mussels form a derived branch (98% support) from a major clade (88% support) that contains the consensus sequence of three intermediate individuals assigned previously as The Bahamas B (Boca Chica) and a second branch (46% support) formed by The Bahamas A, The Bahamas C (Cape Verde) and *B. puniceus* (Figure 3). The few intermediate mussels found in Boca Chica Key by Lee and Foighil (2004, 2005) were most closely related to Fernando de Noronha mussels (*d* = 0.024 ± 0.008), suggesting being introgressed individuals from Fernando de Noronha. The haplotype network (Figure 5), constructed using all the members of Antilles and The Bahamas Clades, showed these individuals to be intermediates between Fernando de Noronha and Antilles B scorched mussels. Fernando de Noronha scorched mussels were situated as a group between *B. puniceus* and The Bahamas, and demonstrated to be a distinct species, suggesting *B. noronhensis,* as a new name for this species.

*Scorched mussels from Pontal Beach of Arraial do Cabo, State of Rio de Janeiro*

Scorched mussels from the canal outlet of Pontal Beach of Arraial do Cabo showed distances over 12% to all *B. exustus* varieties (*16S*: *d* = 0.163 ± 0.021, *COI*: *d* = 0.164 ± 0.023 to Salvador and Cartagena *B. exustus*) and showed to be conspecific with *B. darwinianus*, which was 100% supported by the *COI* ML tree (Figure 3), showing a distance of 0.002 ± 0.001 to the Uruguay population of *B. darwinianus*. Scorched mussels from the canal outlet of Pontal Beach showed four haplotypes at *16S locus* with one most common haplotype in high frequency (N = 10, *f*_*PB-H1*_ = 0.700); and three haplotypes at *COI locus*, showing two most common haplotypes (N = 10, *f*_*PB-CH1*_ = 0.625 and *f*_*PB-CH2*_ = 0.250). The sympatric mussel *M. solisianus* was distinct morphologically from the other mussels and showed large molecular distance from *B. darwinianus* (*16S*, 0.180 ± 0.022; *COI*, 0.151 ± 0.024), from *B. exustus* of Salvador/Cartagena (*16S*, 0.154 ± 0.020; *COI*, 0.158 ± 0.026), and *B. noronhensis* (*16S*, 0.147 ± 0.020; *COI*, 0.180 ± 0.027). The sequences of the exemplars analyzed were deposited in GenBank (Table S1). Although both *Mytilaster* species were clustered in a major branch with *B. darwinianus*, the support of the branch was very low (Figure 3).

*Shell morphology analysis of the Brazilian nominal B. exustus*

**Figure 6 -:**
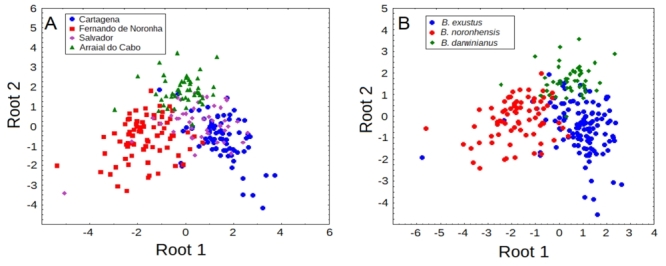
Canonical discriminant space showing the dispersion of scorched mussels based on relative shell parameters: A) scorched mussels allocated to their respective localities, B) scorched mussels allocated to their respective species.

The largest scorched mussels were found in Pontal Beach canal outlet, Arraial do Cabo (N = 45, 23.6 ± 2.6 mm), followed by Salvador (N = 45, 18.6 ± 5.8 mm) which showed sizes similar to Cartagena mussels (N = 75, 19.1 ± 3.1 mm). Fernando de Noronha mussels showed the smallest mean size (N = 75, 11.0 ± 1.9 mm). The CDA multivariate analysis of the relative shell parameters was significant (Wilks’ lambda = 0.19902; *F*(12,632) = 44.323; *p* < 0.0000) and revealed that three of the relative variables were significantly informative for discriminating between groups (rH, *F* = 5.77, *p* = 0.00079; rW, *F* = 88.13, *p* = 0.00000; and rDAL, *F* = 46.50, *p* = 0.00000), from which rW showed the highest correlation with root 1 (*r* = -0.776599) and rDAL with root 2 (*r* = -0.794894). When using classification functions to allocate scorched mussels to their respective localities, only 76% were correctly classified to the respective locality (Figure 6A). When scorched mussels were grouped into their respective species (Salvador and Cartagena to *B. exustus,* Pontal Beach to *B. darwinianus,* and Fernando de Noronha as *B. noronhensis*) correct classification increased to 87% (Figure 6B). *B. noronhensis* appears distinct from *B. exustus* and *B. darwinianus* over root 1 and *B. darwinianus* is distinguished from these two species over root 2 (Figure 6B).

## Discussion

In this study, we partially clarified the distribution of *B. exustus* complex in Brazil by studying populations previously registered as *B. exustus*. In the oceanic island of Fernando de Noronha, we found that scorched mussels differ significantly from the Atlantic and Gulf of Mexico clades of *B. exustus*, being a species more closely related to *B. puniceus* from Cape Verde from The Bahamas Clade. Because it shows an independent evolutionary life since at least the Pleistocene, we propose the new name of *Brachidontes noronhensis*. Scorched mussels found in the state of Rio de Janeiro, associated with brackish and freshwaters, were identified as *B. darwinianus*, which is also found in Argentina and Uruguay and was highly distant from the Atlantic and Gulf of Mexico clades of *B. exustus.* This finding supports the idea of the absence of *B. Exustus* in the southeast of Brazil. We found *B. exustus* in Salvador, State of Bahia (Northeast of Brazil), represented by the Atlantic Clade, and showed high similarity to members distributed north of the Amazon Barrier. This confirmed that the Amazon outflow is a selective barrier to these mussels.

Fernando de Noronha archipelago emerged in the Miocene, 8-12 Myr, due to successive volcanic eruptions during the splitting of the South American and African tectonic plates (Almeida, 1955; Cordani, 1970). Mollusc fauna was characterized to be composed mainly of Antillean forms (Lopes and Alvarenga, 1955). *Brachidontes exustus* was cited in the archipelago by Lopes and Alvarenga (1955), Rios and Barcellos (1979), and Rios (1994). Nonetheless, the Fernando de Noronha population showed large nucleotide distances from *B. exustus* from Salvador (*16S*, 16.6%, and *COI*, 13.9%) and to other *Brachidontes* species (> 19%), characterizing interspecific levels of differentiation (see Hebert et al. 2003). We found Fernando de Noronha scorched mussels closely related to *B. puniceus* from Cape Verde archipelago, which was described as part of The Bahamas Clade of *B. exustus* (Lee and Foighil, 2005). The nucleotide differentiation between Fernando de Noronha population and *B. puniceus* (4.4%) is over the range of intraspecific divergence given for many different taxa (< 2.0% in vertebrates, Avise and Walker, 1999; 0.17-0.33% in Hexapoda, Lepidoptera by Hebert *et al*., 2003; < 2.5% in conoidean gastropod Mollusks, Puillandre et al., 2009; < 3% in Opisthobranch gastropod Mollusks, Malaquias and Reid, 2009; < 2.2% in Ostreidae Bivalves, Liu et al., 2011). Audzijonyte et al. (2012), studying deep-sea clam species delimitation, concluded that values under 5% must be considered distinct species, and divergence above 1.5-2% should be treated as a possible indication of speciation. It was estimated that Fernando de Noronha scorched mussels (*B. noronhensis*, new name) diverged from *B. puniceus* about 1.8-1.1 Myr, demonstrating an independent evolutionary history dating back to the end of the Pliocene and the beginning of the Pleistocene. Speciation in *Brachidontes* is expected to occur primarily through post-recruitment ecological factors (Lee and Foighil, 2005; Trovant et al., 2016) rather than through oceanographic barriers, due to the high larval dispersal capability of scorched mussels (Fuller and Lutz, 1989; Barber et al., 2005; Goto et al., 2011; Morton, 2012). Intermediate individuals found in Boca Chica (named by Lee and Foighil as The Bahamas B clade), may represent introgressed individuals descended from dispersed mussels from Cape Verde and the Fernando de Noronha. Tropical island environments, such as Cape Verde and Fernando de Noronha archipelagos, are considered true hotspots due to their high diversity (Santana et al., 2018). These archipelagos show high endemism resulting from their geographical isolation, and the colonization of these remote habitats generally depends on accidental colonization events, exhibiting a partial sample of continental biodiversity (Cunha et al., 2011). Environmental conditions similar to their origin may also favor the permanence of new settlers. *B. Puniceus* from Cape Verde also extends on the African continent along the western coast from mauritania to Angola (Cunha *et al*., 2011; Morton, 2012). The Cape Verde archipelago is older than Fernando de Noronha, emerging 20-8 Myr (Pim et al., 2008). The Bahamas A Clade to which *B. puniceus*, and now *B. noronhensis* belong, diverged from the Antillean sister clade about 3.4-4.6 Myr (Lee and Foighil, 2005). Rocky-shore species were considered almost immune to extinctions during sea-level change periods (Williams and Reid, 2004), allowing dispersal from the tropics. Our finding adds evidence to the past amphi-Atlantic distribution of The Bahamas A Clade. This supports the hypothesis of an internal marine connection in the past between the Caribbean Sea and the Southern Atlantic, mediated by sea-level variations, supported by the Miocene fossil deposits of *B. puniceus* found in Quail island (Pim *et al*., 2008).

The southeast coast of Brazil receives waters from the South Brazil current and the Subtropical Gyre (Mulitza et al., 2007) (Figure 1). There, *B. exustus* was cited for Arraial do Cabo based on morphological identification (Quintanilha, 2017). Trovant et al. (2013, 2016) studying the southern coast of Brazil, Uruguay and Argentina, using molecular markers to identify species, did not find *B. exustus*. The molecular identification of the Arraial do Cabo population of scorched mussels associated with brackish waters indicated that it belongs to *B. darwinianus.* This species was studied by Trovant *et al.* (2016), enhancing the uncertain origin of this species, with a possible origin from the Caribbe. The species, remained restricted to a southern distribution and evoluted to low salinity tolerance after the amazon barrier emerged (Trovant *et al*., 2016). The dominant rocky-seashore *M. solisianus* coexists with *B. darwinianus* (Tanaka, 2005; Trovant *et al*., 2016) and were clustered together in the ML tree, although with high genetic distance between them and low support of the branch. This uncertainty and the results of Trovant *et al.* (2016) reinforce the need to study the generic status of *M. solisianus* in more detail, which was not the objective of this study.

Scorched mussels from Salvador, State of Bahia, showed a distance of 0.1% to *B. exustus* from Cartagena, Colombia, proving to be conspecific according to molecular taxonomic delimitations indicated by Hebert et al. (2003). The Salvador population belongs to Lee and Foighil (2004, 2005)’ Atlantic Clade of *B. exustus*, which split from the Gulf Clade about 2.2-2.9 Myr during the Pliocene after Panama closure (Lee and Foighil, 2005). Trovant et al. (2016) suggested that in the middle of the Miocene, *B. exustus* expanded its distribution southward, but with the beginning of the Amazon River outflow in the Late Miocene, a parapatric vicariant process divided the *B. exustus* population between the Caribbean and Brazilian populations. 

The Amazon River plume releases a large volume of fresh water into the Atlantic, alters salinity, and causes sediment discharge up to 200-500 km along the shelf from the river mouth (Curtin, 2003). Nonetheless, *B. exustus* extends to Salvador*,* overpassing about 3,000 km south of the Amazon River barrier. In addition, the warm coastal sea-surface North Brazil Current (Muller-Karger et al., 2017) is acting northward through the north coast of South America (Figure 1). This current has flowed in this direction since the Late Miocene (Kirillova et al., 2019), through the Pliocene (Karas et al., 2017) and Pleistocene (Mulitza et al., 2007). This makes it difficult to understand how this population extended its distribution from the Caribbean to the south of the Brazilian Marine Biogeographic region. However, Salvador may represent or be close to the southern limit of *B. exustus*. Warm waters arriving from the cross-equatorial flux of the South Equatorial Current (Garzoli and Matano, 2011; Muller-Karger *et al.*, 2017) (Figure 1) may have maintained the population on the coast of the Brazilian Biogeographic Province since its expansion in the Middle Miocene. The high similarity between Salvador and Cartagena scorched mussels corroborates the present genetic interchange. 

Anthropogenic activities, such as maritime traffic routes between commercial and tourist maritime ports might be a source of interchange in both directions. Ships can transport mussels over large distances, either as adults attached to the hulls or as larvae within ballast water (Sierra-Marquez et al., 2018). An alternative hypothesis is that the Amazon River outflow was not a barrier for species with long-lived larval stages. Other mollusc species were not affected by the Amazon Barrier (Williams and Reid, 2004; Malaquias and Reid, 2009). Trovant et al. (2016) recognized that the Amazon Barrier in its present form might be permeable for scorched musssels. Williams and Reid (2004) suggested that species with a high potential for larval dispersal are not limited by oceanographic barriers maintaining remarkable long-term allopatry. Consistent with Silva et al. (2005), the open-sea extension of the low salinity waters of the Amazon plume varies seasonally from 140-310 km at a depth of 10-20 m; the Brazilian North Current at the maximum Amazon outflow remains narrow and restricted to the talud region of the Amazon Continental Platform and runs wide (horizontally and vertically) and close to the coast during the transition and low outflow periods. These authors also found that the tropical waters of high salinity flow under the low salinity plume during these periods, allowing larvae sensitive to low salinity to cross the Amazon Barrier through the high salinity layer, ultimately dispersing in the south.

Scorched mussels were first studied using the anatomy of soft parts (Avelar and Narchi, 1894a,b; Morton, 2012) and shell morphology (Seed, 1980; Aguirre et al., 2006; Van der Molen et al., 2012; Adami et al., 2013). Most studies that supported *B. exustus* as a widespread species were based on shell variation, considered a result of morphological plasticity to habitat adaptation (Rios, 1994; Aguirre *et al*., 2006). Although morphological approach have been consistent with *COI* genetic distances in other molluscs (Puillandre et al., 2009), we cannot apply for the identification of *Brachidontes* species. The three species identified here by molecular markers (*B. exustus*, *B. noronhensis,* and *B. darwinianus*) were largely distinguished by two morphological parameters (rW and rDAL), although not enough for species identification.

## Supplementary material

The following online material is available for this article:

Table S1 - GenBank accession numbers from species used and obtained in this study.
