# Supplementary material for: Molecular and morphometric analysis of nominal Brachidontes exustus (Mollusca, Mytilidae) in Brazilian waters
Source: Genet Mol Biol. 2022 Apr 29;45(2):e20210247. doi: 10.1590/1678-4685-GMB-2021-0247 (PMC9059129; doi:10.1590/1678-4685-GMB-2021-0247)
Supplement: Table S1 - [file 1415-4757-GMB-45-2-e20210247-s1.pdf]

Genetics and Molecular Biology
https://doi.org/10.1590/1678-4685-GMB-2021-0247

Supplementary Material to "Molecular and morphometric analysis of nominal *Brachidontes exustus* (Mollusca, Mytilidae) in Brazilian waters"

Table S1 - GenBank accession numbers from species used and obtained in this study.

Species	Locality	GenBank Access Number (Authors)
B. rodriguezii	Buenos Aires, Argentina	KC844454 **(1)**
B. rodriguezii	Mar del Plata, Argentina	KY454053 **(2)**
B. darwinianus	Punta del Este, Uruguay	KC844414 **(1)**
B. puniceus	São Vicente Island, Calhau, Cape Verde	HM999789 **(3)**
B. modiolus	Long Key, FL, USA	AY621917 **(5)**
B. modiolus	Bolivar, Colombia	JN991296 **(4)**
B. exustus (The Bahamas A Clade)	Boca Chica Key, FL, USA)	AY621855 **(5)**
B. exustus (The Bahamas B Clade)	Boca Chica, FL, USA	AY621858 **(5)**
B. exustus	Layton, FL, USA	MH012212 **(6)**
B. exustus	USA	KY454042 **(2)**
Geukensia demissa	North West Atlantic	U56844 **(7)**
Geukensia demissa	Osprey Park, NY, USA	MT192779 **(8)**
Geukensia demissa	York River, VA, USA	KU905753 **(9)**
Geukensia demissa	Hudson River, NY, USA	MH012213 **(6)**
Mytilus edulis	Nova Scotia, Canada	KF644321 **(10)**
Mytilus edulis	Hudson Bay, Manitoba, Canada	KF644088 **(10)**
B. exustus complex	Atlantic Clade	AY621861, AY621879, AY621901–AY621908, AY825139, AY825140, AY825204 – AY825206 **(5, 11)**
B. exustus complex	Gulf Clade	AY621866 – AY621878, AY621880–AY621900, AY621910, AY621912–AY621915 **(5, 11)**
B. exustus complex	Bahaman and Antillean clades	AY621835, 838–839, 841, 845, 847–850, 852, 854–855, 858, 909, 911; AY825105, 118–120, 142, 150, 152, 155–156, 188–189, 194–197, 200, 202–203, 207–208, 211, 215 **(5, 11)**
B. noronhensis, new name	Fernando de Noronha archipelago, Brazil	**16S:** MW286328 (FN-H1), MW286329 (FN-H2),; **COI:** MW548973 (FN-CH1), MW548975 (FN-CH2), MW548980 (FN-CH3), MW548971 (FN-CH4), MW548974 (FN-CH5) **(Present study)**
B. exustus (Atlantic Clade)	Salvador, State of Bahia, Brazil	**16S:** MW281510 (SV-H1), MW281511 (SV-H2), MW281512 (SV-H3); **COI:** MW548977 (SV-CH1), MW548967 (SV-CH2), MW548968 (SV-CH3), MW548979 (SV-CH4), MW548972 (SV-CH5) **(Present study)**
B. exustus (Atlantic Clade)	Cartagena, Colombia	**16S:** MW281513 (CT-H1), MW281514 (CT-H2), MW281509 (CT-H3); **COI:** MW548976 (CT-CH1), MW548978 (CT-CH2), MW548969 (CT-CH3) **(Present study)**
B. darwinianus	Pontal Beach, Arraial do Cabo,	**16S:** MW281805 (PB-H1), MW281806 (PB-H2),

Species	Locality	GenBank Access Number (Authors)
	State of Rio de Janeiro, Brazil	MW281808 (PB-H3), MW281807 (PB-H4); **COI:** MW548966 (PB-CH1), MW54896470 (PB-CH2), MW5489 (PB-CH3) (**Present study**)
Mytilaster solisianus	Anjos Beach, Arraial do Cabo, State of Rio de Janeiro, Brazil	**16S:** MW264488 (AB-H1), MW265435 (AB-H2); **COI:** MW548965 (AB-CH1) (**Present study**)
Mytilaster minimus	Mediterranean Sea	DQ836022 **(12)**

(1) Trovant *et al*., 2013; (2) García-Souto *et al*., 2017; (3) Cunha *et al*., 2011; (4) Martinez-Ortega and collaborators, 2011, unpublished; (5) Lee and Foighil, 2004; (6) Metzger *et al*., 2018; (7) Hoeh *et al*., 1998; (8) Reid and collaborators, unpublished; (9) Aguilar and collaborators, unpublished; (10) Layton *et al*., 2014; (11) Lee and Foighil, 2005; (12) Terranova *et al*., 2007.

References

Cunha RL, Lopes EP, Reis DM and Castilho R (2011) Genetic structure of *Brachidontes puniceus* populations in Cape Verde Archipelago shows signature of expansion during the last glacial maximum. J Mollus Stud 77:175-181.

García-Souto D, Sumner-Hempel A, Fervenza S, Pérez-García C, Torreiro A, González-Romero R, Eirín-López JM, Morán P and Pasantes JJ (2017) Detection of invasive and cryptic species in marine mussels (Bivalvia, Mytilidae): A chromosomal perspective. J Nat Conserv 39:58-67.

Hoeh WR, Black MB, Gustafson R, Bogan AE, Lutz RA and Vrijenhoek RC (1998) Testing alternative hypothesis of neotrigonia (Bivalvia: Trigonioida) phylogenetic relationships using cytochrome *c* oxidase subunit I DNA sequences. Malacologia 40:267-278.

Layton KK, Martel AL and Hebert PDN (2014) Patterns of DNA barcode variation in Canadian marine molluscs. PLoS One 9:e95003.

Lee T and Foighil DO (2004) Hidden Floridian biodiversity: Mitochondrial and nuclear gene trees reveal four cryptic species within the scorched mussel, *Brachidontes exustus*, species complex. Mol Ecol 13:3527-3542.

Lee T and Foighil DO (2005) Placing the floridian marine genetic disjunction into a regional evolutionary context using the scorched mussel, *Brachidontes exustus*, species complex. Evolution 59:2139-2158.

Metzger MJ, Paynterd AN, Siddalld MS and Goff SP (2018) Horizontal transfer of retrotransposons between bivalves and other aquatic species of multiple phyla. Proc Natl Acad Sci U S A 115:E4227-E4235.

Terranova MS, Brutto S, Arculeo M, and Mitton JB (2007) A mitochondrial phylogeography of *Brachidontes variabilis* (bivalvia: mytilidae) reveals three cryptic species. J Zool Syst Evol Res 45:289-298.

Trovant B, Ruzzante DE, Basso NG and Orenzans JM (2013) Distinctness, phylogenetic relations and biogeography of intertidal mussels (*Brachidontes*, Mytilidae) from the south-western Atlantic. J Mar Biol Assoc UK 93:1843-1855.
